# Predictive value of low testosterone concentrations regarding coronary heart disease and mortality in men and women – evidence from the FINRISK97 study

**DOI:** 10.1111/joim.12943

**Published:** 2019-06-14

**Authors:** T. Zeller, S. Appelbaum, K. Kuulasmaa, T. Palosaari, S. Blankenberg, P. Jousilahti, V. Salomaa, M. Karakas

**Affiliations:** ^1^ Clinic of General and Interventional Cardiology University Heart Center Hamburg Hamburg Germany; ^2^ German Center for Cardiovascular Research (DZHK) Partner Site Hamburg Lübeck, Kiel, Hamburg Germany; ^3^ National Institute for Health and Welfare Helsinki Finland

**Keywords:** biomarker, coronary heart disease, mortality, prognosis, testosterone

## Abstract

**Introduction:**

The relevance of low testosterone concentrations for incident coronary heart disease (CHD) and mortality has been discussed in various studies. Here, we evaluate the predictive value of low baseline testosterone levels in a large population‐based cohort.

**Methods:**

We measured the serum levels of testosterone in 7671 subjects (3710 male, 3961 female) of the population‐based FINRISK97 study.

**Results:**

The median follow‐up (FU) was 13.8 years. During the FU, a total of 779 deaths from any cause, and 395 incident CHD events were recorded. The age‐adjusted baseline testosterone levels were similar in subjects suffering incident events during FU and those without incident events during FU (men: 15.80 vs. 17.01 nmol L^−1^; *P* = 0.69, women: 1.14 vs. 1.15 nmol L^−1^; *P* = 0.92). Weak correlations of testosterone levels were found with smoking (*R* = 0.09; *P* < 0.001), HDL cholesterol levels (*R* = 0.22, *P* < 0.001), systolic blood pressure (*R* = −0.05; *P* = 0.011), BMI (*R* = −0.23; *P* < 0.001) and waist‐hip‐ratio (*R* = −0.21; *P* < 0.001) in men, and with eGFR (*R* = −0.05; *P* = 0.009) in women. Kaplan–Meier analyses did not reveal a positive association of testosterone levels with incident CHD or mortality. Accordingly, also in Cox regression analyses, testosterone levels were not predictive for incident CHD or mortality – neither in men (HR 1.02 [95%CI: 0.70–1.51]; *P* = 0.79 for lowest versus highest quarter regarding CHD and HR 1.06 [95%CI: 0.80–1.39]; *P* = 0.67 regarding mortality), nor in women (HR 1.13 [95%CI: 0.69–1.85]; *P* = 0.56 for lowest versus highest quarter regarding CHD and HR 0.99 [95%CI: 0.71–1.39]; *P* = 0.80 regarding mortality).

**Conclusions:**

Low levels of testosterone are not predictive regarding future CHD or mortality – neither in men, nor in women.

## Graphical abstract

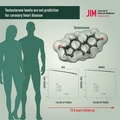

## Introduction

The relevance of low testosterone concentrations has been evaluated in multiple studies 1, 2. The volume of evidence that links low testosterone levels with coronary heart disease (CHD) and mortality has been steadily growing during the last two decades 3. Consistently, rates of testosterone therapy are increasing although the effects of this therapy on cardiovascular outcomes and mortality are unknown 4, 5.

Male sex is considered a strong risk factor for coronary artery disease, and it is widely accepted that men experience a gradual decline in testosterone levels with increasing age 6, 7. Likewise, in the Massachusetts male ageing study, circulating testosterone levels were highest around the age of 30, and subsequently declined at a rate of 1 to 1.5 per cent annually 8. This has prompted multiple investigators to search for a potential link between circulating testosterone levels and CHD. Various studies suggested that testosterone deficiency increases rates of CHD and mortality by adverse effects on insulin resistance, arterial blood pressure, lipid status, weight and visceral obesity, but also by activating inflammatory pathways resulting in higher levels of C‐reactive protein, interleukin‐6 and tumour necrosis factor‐α 9.

Although multiple studies proposed such prognostic effect of low testosterone levels on CHD and mortality, the vast majority of these studies suffer from various limitations and methodological shortcomings 3. Studies showing a significant association between testosterone levels and incident CHD usually suffered from small sample size, limited follow‐up and incomplete statistical adjustment 3, whereas studies that were unable to show a significant association were usually larger and had a longer follow‐up 3. Most recently, a new study from the Framingham heart study reported on the cross‐sectional association between circulating testosterone and cardiac MRI measures of myocardial mass, structure and function 10. Pencina and co‐workers showed that testosterone values were not independently associated with myocardial mass and function after adjustment for established cardiovascular risk factors 10.

Therefore, in the light of the conflicting reports, within this study, we aimed to provide more evidence on the potential role of low serum testosterone levels for future CHD events and mortality.

## Methods

All analyses and biomarker measurements presented were performed within the frame of the BiomarCaRE consortium 11.

### Study population

The present study is based on individuals from the FINRISK97 study aged 25–74 years drawn from the national Finnish population register in 1997. The design of the FINRISK97 study has been published elsewhere 12, 13. This prospective population‐based study was carried out in five districts: North Karelia, Northern Savo (former Kuopio Province), Southwestern Finland, Oulu Province and the region of Helsinki and Vantaa. In total, 11 500 individuals were invited and 8444 (73%) participated in the clinical examination. During the follow‐up period of up to 15 years, the follow‐up rate was 100% for the participants who continued living in Finland. Those who had permanently moved abroad (0.5% of the participants prior to Dec. 31st, 2011) were lost to follow‐up. All individuals enrolled in the study received a physical examination and a self‐administered questionnaire, and a blood sample was drawn. Prior to drawing the blood samples, the individuals were asked for a 4‐h fasting period, avoiding heavy meals during the day. The median fasting time was 5 h with an interquartile range of 3–7 h. The blood samples were stored under standardized conditions at −70°C. For the present paper, we excluded women who were pregnant at the time of the clinical examination, individuals on testosterone‐supplementation therapy and individuals with prevalent cancer or CHD. Our analysis is therefore based on 7671 (49.4% men and 50.6% women) individuals. The Ethics Committee of the National Public Health Institute approved the study, which followed the Declaration of Helsinki. All subjects gave written informed consent.

### Outcome information

The National Hospital Discharge Register, the National Causes of Death Register and the National Drug Reimbursement Register were used to identify fatal and nonfatal end‐points during follow‐up. Incident CHD comprised first myocardial infarction, coronary death, hospitalized unstable angina pectoris and any coronary revascularization (percutaneous transluminal coronary angioplasty or coronary bypass surgery). The definition of the end‐points was according to MORGAM criteria 14. The use of Finnish national healthcare registries for identifying these cardiovascular outcomes has recently been validated 15.

### Laboratory methods

Blood samples were stored under standardized conditions at −70°C. Routine laboratory parameters were measured at the Disease Risk Unit in the National Institute for Health and Welfare, Helsinki. Testosterone levels were measured at the BiomarCaRE/MORGAM Laboratory, University Heart Center Hamburg, Germany, using a chemiluminescent microparticle immunoassay (CMIA) (Abbott ARCHITECT 2nd Generation Testosterone; Abbott Diagnostics). The assay range was 0.45–35 nmol L^−1^, the intra‐assay coefficient of variation (CV) was 3.57%, and the inter‐assay CV was 8.83%. Testosterone levels below or above the assay range were assigned as follows: For levels below 0.45 nmol L^−1^, 0.45 nmol L^−1^ was used, and for levels reported to be above than 35.0 nmol L^−1^, 35.0 nmol L^−1^ was used. The estimated glomerular filtration rate (eGFR) was calculated using the CKD‐Epi equation 16.

### Statistical methods

Multiple imputations were used to manage missing values 17. Individuals with prevalent CHD were excluded from all analysis. Baseline characteristics are presented as percentages for dichotomous variables and as means and standard deviations (SD) for continuous variables. In case, the distribution was symmetric, and mean and standard deviation were given. In case, the distribution was skewed, and median and interquartile ranges (IQR) were preferred. Pearson correlation coefficients were calculated. Additionally, age‐adjusted Pearson correlations were calculated. Age‐adjusted Kaplan–Meier curves for CHD and mortality were produced using categorized (by quartiles) testosterone concentrations. To examine the association of testosterone with CHD and mortality, Cox regression models with age as the timescale were used. Each sex was considered separately due to the different shape of the testosterone distributions of men and women. Cox regression was performed with testosterone levels as a continuous and a categorized variable. Continuous testosterone levels were log transformed for women and left untransformed for men. Two different adjustments were used. Model 1 adjusted only for region of Finland (east, west), whilst model 2 additionally adjusted for log‐transformed total cholesterol, log‐transformed HDL cholesterol, log‐transformed systolic blood pressure, hypertension, diabetes, current smoking, waist–hip ratio, BMI and time of day of blood draw, since testosterone values undergo circadian rhythm 18. R version 3.2.2 (R Foundation for Statistical Computing, Vienna, Austria) was used for all analyses. All tests were two‐tailed, and *P* < 0.05 was considered statistically significant.

## Results

**Figure 1:**
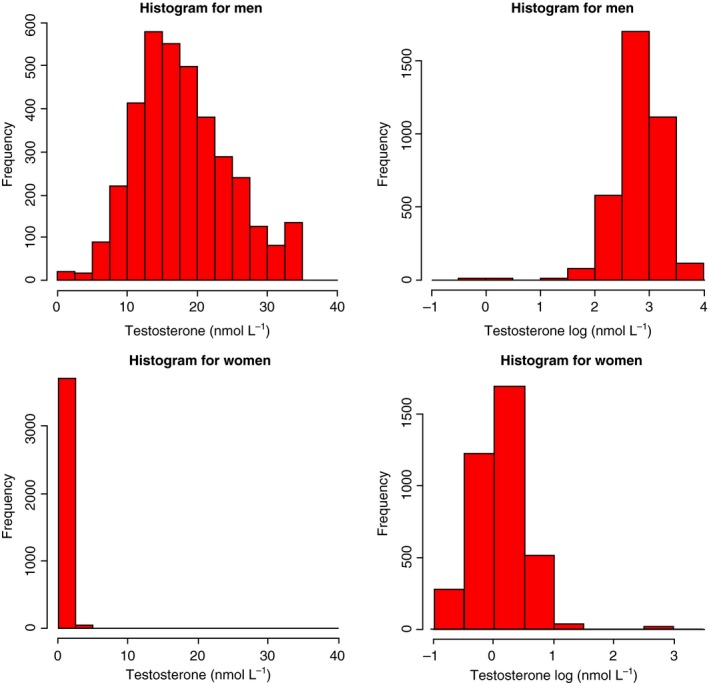
Distribution of baseline serum testosterone levels in men and in women.

The baseline characteristics of the study participants are presented in Table 1. The mean age of the participants was 48.2 years for men and 46.9 years for women. In general, men had a higher prevalence of cardiovascular risk factors than women. As shown in Table 2, testosterone levels were at a median of 17.01 (SD: 9.20) nmol L^−1^ in men and at 1.15 (SD: 0.69) nmol L^−1^ in women (*P* < 0.001). Figure 1 provides the distribution of testosterone levels in men and women.

**Table 1:** Characteristics of study participants at baseline

	Men	Women
*n*	3710	3961
Age [years] (SD)	48.2 (22.6)	46.9 (21.0)
BMI [kg m^−2^] (SD)	26.5 (4.8)	25.5 (6.3)
Current smoker [%]	26.6	17.4
Diabetes [%]	5.2	4.9
Hypertension [%]	16.6	13.6
HDL‐C [mmol L^−1^] (SD)	1.24 (0.39)	1.51 (0.47)
Total cholesterol [mmol L^−1^] (SD)	5.5 (1.4)	5.4 (1.4)
Systolic blood pressure [mmHg] (SD)	136 (25)	129 (27)
Testosterone [nmol L^−1^] (SD)	17.01 (9.20)	1.15 (0.69)

BMI, body mass index; HDL‐C, high‐density lipoprotein cholesterol; SD, standard deviation.

**Table 2:** Distribution of baseline serum testosterone levels (nmol L^−1^) according to gender

	Quarter 1	Quarter 2	Quarter 3	Quarter 4
Men	0; 12.82	12.82; 17.01	17.01; 21.99	21.99; 35.0
Women	0; 0.87	0.87; 1.15	1.15; 1.56	1.56; 35.0

Values are shown as normalized median values.

To assess the correlation of testosterone levels with clinical variables, Pearson correlation coefficients were calculated (Table 3). Age‐adjusted Pearson analyses revealed rather weak, however statistically significant correlations of testosterone levels with smoking (*R* = 0.09; *P* < 0.001), HDL cholesterol levels (*R* = 0.22, *P* < 0.001), systolic blood pressure (*R* = −0.05; *P* = 0.011), BMI (*R* = −0.23; *P* < 0.001) and waist–hip ratio (*R* = −0.21; *P* < 0.001) in men, and with eGFR (*R* = −0.05; *P* = 0.009) in women. Surprisingly, age itself showed a very weak correlation with baseline testosterone levels (*R* = 0.03; *P* = 0.16 in men, *R* = 0.04; *P* = 0.034 in women).

**Table 3:** Age‐adjusted Pearson correlation coefficients of serum testosterone levels with clinical variables

	Men R, *P*‐value	Women R, *P*‐value
Age	0.03; 0.16	0.04; 0.034
Smoking	0.09; <0.001	−0.01; 0.77
Total cholesterol	−0.01; 0.71	−0.01; 0.45
HDL‐C	0.22; <0.001	−0.03; 0.095
Systolic blood pressure	−0.05; 0.011	0.03; 0.055
eGFR	<0.009; 0.82	−0.05; 0.009
Testosterone (log)	0.87; <0.001	0.89; <0.001
BMI	−0.23; <0.001	0.03; 0.085
WHR	−0.21; <0.001	0.02; 0.23

BMI, body mass index; eGFR, estimated glomerular filtration rate; HDL‐C, high‐density lipoprotein cholesterol; R, correlation coefficient; WHR, waist‐to‐hip ratio.

**Figure 2:**
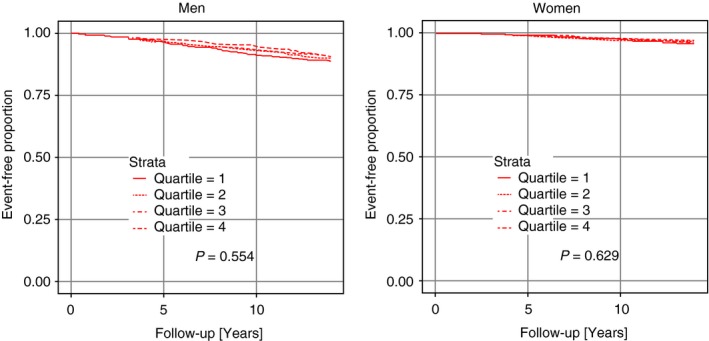
Kaplan–Meier curves for the end‐point incident CHD according to quarters of testosterone.

**Figure 3:**
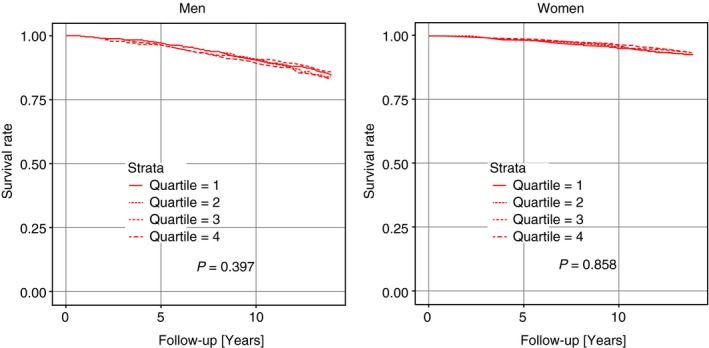
Kaplan–Meier curves for the end‐point all‐cause mortality according to quarters of testosterone.

During a median follow‐up of 13.8 years, a total of 395 incident CHD cases (5.2%) were recorded (275 male, 120 female) and 779 deaths occurred (510 male, 269 female). Baseline testosterone levels were similar in subjects who experienced CHD events or death during follow‐up (i.e. ‘cases’) and in subjects who did not experience CHD events or death during follow‐up (i.e. ‘noncases’). Levels were 16.00 vs. 17.01 nmol L^−1^ in men (*P* = 0.39), and 1.17 vs. 1.15 nmol L^−1^ in women (*P* = 0.44). Kaplan–Meier analyses in men as well as in women (Fig. 2) did not reveal a significant association between CHD incidence and testosterone level. Similarly, Kaplan–Meier analyses showed that low levels of testosterone were not prognostic for all‐cause mortality (Fig. 3). Consistently, as shown in Tables 4 and 5, neither basic nor multivariate adjusted Cox regression showed a significant association between testosterone levels and incident CHD or mortality in the fully adjusted model 2, both in men (HR 1.02 [95%CI: 0.70–1.51]; *P* = 0.79 for lowest versus highest quarter regarding CHD and HR 1.06 [95%CI: 0.80–1.39]; *P* = 0.67 regarding mortality) and in women (HR 1.13 [95%CI: 0.69–1.85]; *P* = 0.56 for lowest versus highest quarter regarding CHD and HR 0.99 [95%CI: 0.71–1.39]; *P* = 0.80 regarding mortality).

**Table 4:** Hazard Ratios (95% CI) of baseline serum testosterone levels with CHD during follow‐up

	Quarter 1 (highest)	Quarter 2	Quarter 3	Quarter 4 (lowest)	*P* for trend
HR (95%CI)					
Men					
Model 1	1	1.32 (0.92–1.90)	1.25 (0.86–1.80)	1.44 (1.00–2.07)	0.075
Model 2	1	1.22 (0.84, 1.75)	0.96 (0.66, 1.40)	1.02 (0.70, 1.51)	0.79
Women					
Model 1	1	0.69 (0.40–1.16)	0.72 (0.44, 1.21)	0.87 (0.55, 1.40)	0.61
Model 2	1	0.79 (0.46, 1.35)	0.90 (0.54, 1.52)	1.13 (0.69, 1.85)	0.56

HR, hazard ratio; CI, confidence interval.

Model 1: age was used as timescale, adjusted for geographical region.

Model 2: additionally adjusted for total cholesterol (log), HDL cholesterol (log), systolic blood pressure (log), known hypertension, known diabetes, smoking status and time period of blood drawn.

**Table 5:** Hazard Ratios (95% CI) of baseline serum testosterone levels with mortality during follow‐up

	Quarter 1 (highest)	Quarter 2	Quarter 3	Quarter 4 (lowest)	P for trend
HR (95%CI)					
Men					
Model 1	1	0.95 (0.73–1.24)	1.06 (0.82–1.36)	1.15 (0.89–1.49)	0.23
Model 2	1	0.94 (0.73, 1.23)	0.98 (0.75, 1.28)	1.06 (0.80, 1.39)	0.67
Women					
Model 1	1	0.88 (0.63–1.23)	0.72 (0.51, 1.02)	0.88 (0.63, 1.21)	0.26
Model 2	1	0.89 (0.63, 1.25)	0.79 (0.56, 1.13)	0.99 (0.71, 1.39)	0.80

HR, hazard ratio; CI, confidence interval.

Model 1: age was used as timescale, adjusted for geographical region.

Model 2: additionally adjusted for total cholesterol (log), HDL cholesterol (log), systolic blood pressure (log), known hypertension, known diabetes, smoking status and time period of blood drawn.

## Discussion

During the recent decade, a wide series of blood‐based biomarkers have been evaluated with regard to cardiometabolic diseases 19, 20, 21, 22, 23, 24, 25, 26, 27, 28, 29, 30, 31. Of these, testosterone has gained special interest, since supplementation is widely used 4. Whilst the relationship of testosterone and CHD has been studied extensively, early studies showed an association of low serum testosterone levels with increased mortality rates in men with and without coronary artery disease, whilst newer studies indicated a nonlinear relationship with increased risk of future ischaemic arterial events at patients with low and high serum levels 32, 33.

In this study, we evaluated the prognostic value of serum testosterone levels for the incidence of CHD and mortality in men and in women. The current data of a large European population failed to demonstrate an association between low testosterone levels and future occurrence of CHD or increased mortality. Our results reflect strict adjustment for covariates, which affect cardiovascular risk and are independently associated with testosterone status. By its strict methodological approach and its prospective design with long‐term follow‐up of almost 14 years and large sample size, as well as the inclusion of women, our study delivers very strong evidence on the irrelevance of low testosterone levels regarding future CHD risk and mortality. These results seem surprising in view of two previous reports of the FINRISK97 study, linking low testosterone levels to incident diabetes and also atrial fibrillation and subsequent stroke in men 1, 2. However, in contrast to CHD, for both entities, distinct testosterone‐based pathomechanisms are well understood. For atrial fibrillation, various experimental studies suggest that systemic hormones alter electrophysiology and electroanatomy in the atrium 1. Testosterone is a potent inhibitor of L‐type calcium channels and blocks the effects of acetylcholine on the atria. Studies in the rat model showed that the immunoreactive protein levels of ryanodine receptor type 2 and sodium‐calcium exchanger significantly increased in orchiectomized male rats as compared with sham‐operated male rats, and orchiectomized male rats with the administration of testosterone 1.

Regarding increased risk of diabetes, this might be mediated by obesity: as shown in Table 3, the strongest (in this case inverse) correlation for testosterone was found for BMI and WHR. Obese men show the enhanced conversion of androstenedione to estrogens by aromatization, which occurs due to adipose tissue‐driven elevated aromatase levels 2. Other studies in rodents and humans documented that metabolic endotoxemia leads to a decline in gonadal function, and that leptin, which is produced by adipose tissue, inhibits testosterone secretion from the Leydig cells 2.

One would expect that elevated rates of diabetes as well as atrial fibrillation/stroke would increase the testosterone‐associated risk for CHD and all‐cause mortality 9, 34. However, those detrimental effects of diabetes and atrial fibrillation/stroke take decades to impair survival and might not be covered by the follow‐up of almost 14 years in the present study.

One of the most striking results in our analysis is that age showed practically no correlation with baseline testosterone levels (*R* = 0.03 in men and 0.04 in women). In part, this might be due to the truncated age distribution, which extended only up to 74 years at baseline. Multiple previous studies indicated that serum testosterone appears to decline as men age 6, 7. Although the described decline has usually been modest, circulating levels have been shown to fall below the normal range of healthy young men, in whom the reference values have been determined 8. In contrast, moderate but statistically significant correlations of testosterone levels in our study were seen with BMI (*R* = −0.23; *P* < 0.001), and waist–hip ratio (*R* = −0.21; *P* < 0.001). However, these correlations were not observed in women, who presented with clearly lower testosterone levels. The negative correlation with BMI/obesity and WHR/increased abdominal adiposity in men is most probably explained by consecutively elevated aromatase levels, which lower the availability of pituitary gonadotrophins and activate the conversion of testosterone to estradiol 35.

### Study limitations

Our study has some limitations that need to be addressed. First, we did not test serial samples. We therefore cannot explore the impact of changes on testosterone concentration towards future disease development. Secondly, we therefore cannot answer whether intra‐individual increasing values (e.g. under supplementation therapy) would impact the future risk of CHD. Moreover, we did not measure other emerging biomarkers, such as CRP, and therefore cannot report respective correlations with serum testosterone. Finally, samples had been stored for about twenty years and degradation processes, which would affect both individuals with and without events, cannot be excluded.

## Conclusion

Low levels of testosterone are not predictive regarding future CHD or mortality – neither in men, nor in women.

## Funding and Conflicts of Interest

This work has been supported by the European Union′s Seventh Framework Programme (FP7/2007‐2013) under grant agreement No. HEALTH‐F2‐2011‐278913 (BiomarCaRE). VS has been supported by the Finnish Foundation for Cardiovascular Research, participated in a conference trip sponsored by Novo Nordisk and received a honorarium for participating in an advisory board meeting. He also has ongoing research collaboration with Bayer Ltd. SB has received research funding from Boehringer Ingelheim, Bayer Healthcare, Abbott Diagnostics, Siemens, Thermo Fisher and Roche Diagnostics and received honoraria for lectures or consulting from Boehringer Ingelheim, Bayer, Roche, Astra Zeneca, Siemens, Thermo Fisher and Abbott Diagnostics. MK has received research funding from Vifor Pharma and received honoraria for lectures or consulting from Astra Zeneca, Amgen, Sanofi‐Aventis and Vifor Pharma. TZ is supported by the German Center for Cardiovascular Research (DZHK, 81Z1710101). All other authors declare no conflict of interest. Drs Karakas and Zeller had full access to all the data in the study and take responsibility for the integrity of the data and the accuracy of the data analysis.
